# Tissue Eosinophilia of the Colonic Mucosa as a Potential Early Predictor of Failure to Achieve Clinical Remission in Ulcerative Colitis

**DOI:** 10.3390/medicina62061024

**Published:** 2026-05-25

**Authors:** Ilona Pak, Elyarbek Tashmetov, Kamila Tolegen, Meirambek Askarov, Dmitriy Klyuyev, Yevgeniy Kamyshanskiy

**Affiliations:** 1 Department of Surgical Diseases, Karaganda Medical University, Karaganda 100000, Kazakhstan; i.pak@qmu.kz (I.P.); tashmetov.e@gmail.com (E.T.); 2 Institute of Life Sciences, Karaganda Medical University, Karaganda 100000, Kazakhstan; tolegenk7997@mail.ru (K.T.); klyuev@qmu.kz (D.K.); 3 Clinic of Medical University, Karaganda Medical University, Karaganda 100000, Kazakhstan; kamyshanskiy84@mail.ru

**Keywords:** ulcerative colitis, eosinophils, colonic mucosa, tissue eosinophilia, extraintestinal manifestations

## Abstract

*Background*: Eosinophilic infiltration is frequently observed in ulcerative colitis (UC), but its impact on disease course remains incompletely understood. This study aimed to assess eosinophilic infiltration of the colonic mucosa in patients with newly diagnosed UC and to investigate its association with the achievement of clinical remission during the first year of follow-up. *Methods*: A retrospective study was conducted in patients with newly diagnosed UC. Patients were stratified into two groups according to clinical outcome during the first year of follow-up: clinical remission (*n* = 30) and non-remission (*n* = 30). Clinical and laboratory data were extracted from an integrated medical information system database. Archived colonic mucosal biopsy specimens were independently evaluated by two pathologists. Mean eosinophil density across five high-power fields and peak eosinophil count were assessed. *Results*: In primary biopsy specimens, the median eosinophil density was 19 (11 to 27) cells in the clinical remission group and 33 (23 to 51) cells in the non-remission group. Logistic regression analysis showed that an increased eosinophil count (OR 6.48; 95% CI 1.76 to 23.88; *p* = 0.005) and the presence of extraintestinal manifestations (OR 5.78; 95% CI 1.17 to 28.6; *p* = 0.031) were associated with failure to achieve clinical remission during the first year of treatment. *Conclusions*: In adult patients with ulcerative colitis, a higher density of eosinophils in the colonic mucosa at the time of initial diagnosis is associated with failure to achieve clinical remission during the first year of treatment. These results should be considered hypothesis-generating and require confirmation in larger prospective studies to further clarify the potential prognostic significance of tissue eosinophilia in ulcerative colitis.

## 1. Introduction

Ulcerative colitis (UC) is a chronic relapsing inflammatory disease of the gastrointestinal tract with a multifactorial and incompletely understood etiology, the global prevalence of which continues to increase [1,2,3]. The primary therapeutic goal in UC management is the achievement and maintenance of sustained long-term clinical remission [4], as updated in a subsequent correction [4]. The disease is characterized by an unpredictable clinical course and a variable response to therapy [5,6], which has stimulated growing interest in potential determinants of persistent inflammation, including the cellular composition of the inflammatory infiltrate.

The prognosis of ulcerative colitis is determined by a combination of clinical, laboratory, endoscopic, and histological parameters. Factors associated with an unfavorable disease course include early age at disease onset, extensive colonic involvement (pancolitis), high endoscopic activity, the presence of extraintestinal manifestations, elevated inflammatory markers, and specific histological features [7,8,9,10].

Eosinophils are multifunctional granulocytic leukocytes traditionally associated with allergic reactions and parasitic infections [11,12]. More recently, they have been increasingly recognized as important modulators of tissue regeneration and immune regulation within the intestinal mucosa [13,14,15]. Accumulating evidence suggests that eosinophils play an active role in the pathogenesis of inflammatory bowel diseases (IBD), including UC [13,16,17,18,19,20,21]. However, the available data on the relationship between eosinophils and disease activity [22,23,24,25,26,27,28,29,30], response to therapy [28,29,30,31], disease outcomes [32,33,34,35], and the risk of clinical relapse in UC [36,37,38,39] remain heterogeneous and, in some cases, contradictory. Specifically, some studies have reported an association between eosinophil levels and relapse frequency, whereas others have not confirmed such a relationship. In addition, eosinophil-predominant inflammation has been shown to be associated with a lower risk of relapse compared with neutrophil-predominant inflammation [36]. In some studies, eosinophilia was associated with a poorer response to initial therapy, whereas low eosinophil density was associated with a more severe disease course requiring treatment escalation. Taken together, these data highlight the complex and ambiguous role of eosinophils in the pathogenesis and clinical course of ulcerative colitis. The uncertain clinical significance of eosinophils is also emphasized in current ECCO clinical guidelines, which state that eosinophils should not be used as a marker of histological activity in ulcerative colitis [40]. In addition, morphometric evaluation is complicated by the lack of unified diagnostic criteria and standardized threshold values for colonic tissue eosinophilia [41,42,43].

Assessment of eosinophilic infiltration in the colonic mucosa at the stage of primary diagnosis of ulcerative colitis also remains an unresolved issue. Early disease characterization often determines the subsequent clinical trajectory and is therefore of particular importance for risk stratification and the development of personalized therapeutic strategies in UC patients. The inconsistency of current evidence regarding the prognostic significance of eosinophils in UC, particularly during the early stage of the disease, highlights the need for further investigation in this area.

The present study was conducted in a cohort of patients with ulcerative colitis from Central Kazakhstan to evaluate the significance of clinical and histological parameters associated with failure to achieve clinical remission during the first year of follow-up after diagnosis.

## 2. Materials and Methods

### 2.1. Study Design

A retrospective cohort study was conducted in patients with newly diagnosed ulcerative colitis (UC) between January 2019 and January 2024 at the clinical site of Karaganda Medical University, Regional Clinical Hospital, Karaganda, Kazakhstan.

Patients were eligible for inclusion if colonoscopy with colonic mucosal biopsy was performed at the time of initial diagnosis and if follow-up endoscopic examinations with histological evaluation of biopsy specimens were available at 6 and 12 months.

The study was conducted in accordance with the ethical principles of the Declaration of Helsinki. The study protocol was approved by the Local Bioethics Commission of the NCJSC Karaganda Medical University (Protocol No. 1, dated 20 September 2022). Written informed consent was obtained from all participants.

To investigate eosinophilic infiltration of the colonic mucosa, patients were stratified into two groups according to the clinical course of the disease during the 12-month follow-up period.

The clinical remission group included 30 patients who achieved clinical remission at both the 6-month and 12-month follow-up visits. Clinical remission was defined as a total Mayo score ≤ 2 points, with no individual subscore exceeding 1 point [44].

The non-remission group included 30 randomly selected patients who continued to demonstrate clinical and/or endoscopic disease activity at the 6-month and/or 12-month follow-up assessments. An additional criterion for inclusion in this group was a decrease in the total Mayo score of at least 2 points during the first year of observation compared with baseline disease activity at the time of initial diagnosis. However, the total Mayo score at 12 months did not exceed 7 points.

The diagnosis of ulcerative colitis was established according to generally accepted national and European clinical, endoscopic, and histological criteria [4]. In all cases, the diagnosis was confirmed by a specialized gastroenterologist and remained unchanged during at least two years of clinical follow-up.

#### 2.1.1. Inclusion Criteria

Patients were eligible if they met the following criteria:

- age ≥ 18 years;
- availability of at least three colonoscopies with biopsy (baseline colonoscopy performed at the time of diagnosis before initiation of therapy);
- complete medical records available for the entire follow-up period.

#### 2.1.2. Exclusion Criteria

Patients were excluded in the presence of:

- co-infection with human cytomegalovirus (CMV) or *Clostridioides difficile*;
- absence of clinical response to therapy, defined as a decrease in the total Mayo score < 2 points within 12 months or persistent high disease activity (Mayo score > 7 after 12 months);
- biological therapy at any stage of follow-up;
- systemic corticosteroid therapy within 30 days before biopsy collection;
- history of colorectal cancer or colectomy;
- loss to follow-up (absence of visits at 6 and 12 months);
- positive stool examination for helminth eggs or parasites;
- active infectious diseases, decompensated cardiovascular diseases, malignant neoplasms, tuberculosis, or HIV infection.

**Figure 1:**
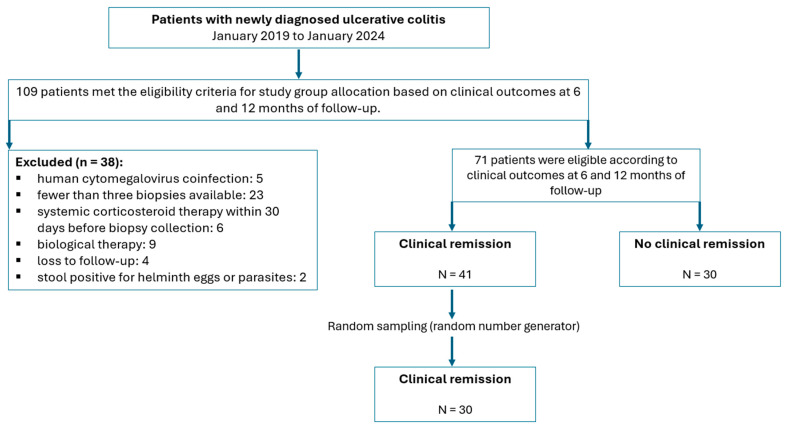
Study design and patient selection flowchart.

From the total cohort of eligible patients, an analytical subcohort for morphometric analysis was generated using random sampling based on a random number generator, ensuring equal distribution according to clinical outcomes. The study design is presented in Figure 1.

### 2.2. Routine Clinical Management

Patients received standard therapy according to the recommendations of the European Crohn’s and Colitis Organisation (ECCO) [45] and the national clinical protocol for the diagnosis and management of ulcerative colitis.

Treatment strategies were aimed at achieving clinical and endoscopic remission and included stepwise escalation of therapy in cases of persistent disease activity. Follow-up endoscopic examinations were performed at 6 and 12 months or earlier when clinically indicated.

### 2.3. Data Collection

Clinical and laboratory data were extracted from the hospital electronic medical information system and subsequently transferred into a secure anonymized database for analysis.

All recorded demographic and clinical characteristics of the patients are summarized in Table 1.

### 2.4. Definitions

A primary biopsy was defined as a biopsy of the colonic mucosa obtained during the initial diagnostic colonoscopy.

Comorbid conditions were assessed for each patient using the Charlson Comorbidity Index [46].

Treatment adherence was defined as regular intake of prescribed medications for ulcerative colitis and scheduled clinical visits [47].

Endoscopic disease activity was assessed using the Mayo Endoscopic Subscore (MES 0–3) [48,49].

Disease extent was classified according to the Montreal classification: E1—ulcerative proctitis; E2—left-sided colitis; E3—extensive colitis (pancolitis) [50].

Histological disease activity was evaluated using the Nancy histological index [51].

Clinical remission was defined as the absence of clinical symptoms of disease activity with a total Mayo score ≤ 2 points and no individual subscore > 1 point [44].

Extraintestinal manifestations of UC were defined as systemic clinical manifestations occurring outside the gastrointestinal tract and associated with immune-mediated mechanisms, metabolic disturbances, or chronic systemic inflammation [40].

### 2.5. Tissue Sampling

Endoscopic examinations were performed according to a standardized biopsy protocol. Biopsy specimens were obtained from areas of maximal inflammatory activity as well as from visually unaffected mucosa.

For the initial diagnosis of ulcerative colitis, at least two biopsy samples were obtained from a minimum of five segments of the colon and the terminal ileum [45]. For the evaluation of disease activity, at least two biopsy samples were collected from the most affected regions of the right and left colon or from the rectosigmoid segment. For dysplasia surveillance, biopsies were obtained from the cecum/ascending colon, transverse colon, descending colon, and rectosigmoid colon.

### 2.6. Histological Processing

Biopsy specimens were fixed in 10% neutral buffered formalin for 24 h, dehydrated in graded ethanol solutions, cleared in xylene, and embedded in paraffin. Paraffin-embedded tissue sections of 3 μm thickness were cut using a microtome and mounted on glass slides. Slides were then deparaffinized and stained.

Tissue sections were stained with Mayer’s hematoxylin for 15 min, followed by washing in running water for 5 min. Subsequently, sections were counterstained with eosin for 1 min.

### 2.7. Morphological and Morphometric Assessment

Eosinophil counts were performed independently by two gastrointestinal pathologists who were blinded to the clinical data, biopsy timing, and patient group allocation. Interobserver agreement for eosinophil quantification was evaluated using the intraclass correlation coefficient (ICC).

Histological slides were first examined under low magnification (×40) to identify areas of maximal eosinophilic infiltration within the lamina propria, excluding crush artifacts, biopsy edges, necrotic areas, blood deposits, and lymphoid follicles. Eosinophils were identified as cells with bilobed nuclei and abundant cytoplasm containing eosinophilic granules [52].

Eosinophil counts were performed in five non-overlapping high-power fields (HPF) using a light microscope (Zeiss Axiolab, Carl Zeiss Suzhou Co. Ltd., Suzhou, China) at ×400 magnification (objective ×40, ocular ×10). One high-power field corresponded to an area of 0.331 mm^2^. Each field was required to be located at least four crypts away from the nearest lymphoid follicle. Intravascular and/or degranulated eosinophils were not included in the analysis. For each case, mean eosinophil density in the colonic mucosa was calculated as the arithmetic mean of the five evaluated fields. When biopsies from multiple colonic segments were available, the highest value among all specimens was used for analysis.

In addition, the peak eosinophil count was assessed quantitatively and defined as the highest eosinophil count observed across the five examined high-power fields. When biopsy specimens from multiple colonic segments were available, the highest peak eosinophil count identified in any segment was used for the primary analysis.

For categorical stratification, previously proposed threshold values were used. Increased eosinophilic infiltration was defined as:

- 50 eosinophils per HPF in the right colon;
- 25 eosinophils per HPF in the left colon [53,54].

### 2.8. Statistical Analysis

Statistical analysis was performed using Statistica 10.0 and IBM SPSS Statistics 27.0 (IBM Corp., Chicago, IL, USA).

Descriptive statistics were used to summarize the data. Quantitative variables were assessed for normality of distribution using the Shapiro–Wilk and Kolmogorov–Smirnov tests. If the *p*-value was <0.05, the null hypothesis of normal distribution was rejected. Normally distributed variables were presented as mean ± standard deviation, whereas non-normally distributed variables were expressed as median with first and third quartiles (Q1–Q3).

Interobserver agreement for quantitative parameters was assessed using the intraclass correlation coefficient (ICC), based on a mixed-effects model with absolute agreement for single measurements. For categorical variables, Cohen’s kappa coefficient (k) was used. Interpretation of the values was performed according to the recommendations of Cicchetti: <0.40, poor reliability; 0.40–0.59, fair reliability; 0.60–0.74, good reliability; and >0.75, excellent reliability [54].

Subsequently, clinical, endoscopic, and histological factors were compared between patients who achieved clinical remission and those who did not using the chi-square test with Yates’ correction (or Fisher’s exact test), as appropriate, or Student’s *t*-test or the Mann–Whitney U test.

Binary logistic regression analysis was performed to evaluate potential predictors of clinical outcomes. For all logistic regression analyses, odds ratios (OR) with 95% confidence intervals (CI) were calculated. Variables with *p* < 0.1 in univariate logistic regression were included in multivariate models. Backward stepwise selection was applied to remove non-significant variables. Internal validation of the model was performed using non-parametric bootstrap resampling (1000 iterations).

All hypothesis testing was performed using two-sided *p*-values, and statistical significance was set at *p* < 0.05.

## 3. Results

### 3.1. Clinical, Laboratory, Endoscopic, and Histological Characteristics of the Study Groups at Baseline

At baseline, extraintestinal manifestations were observed in 3 patients (10.0%) in the clinical remission group and in 12 patients (40.0%) in the non-remission group. In the clinical remission group, 2 patients (66.7%) had autoimmune manifestations associated with disease activity, including arthritis and aphthous stomatitis, whereas 1 patient (33.3%) had autoimmune manifestations not associated with disease activity, namely primary sclerosing cholangitis and psoriasis. In the non-remission group, 4 patients (33.3%) had autoimmune manifestations associated with disease activity, 5 patients (41.7%) had autoimmune manifestations not associated with disease activity, and 3 patients (25.0%) had complications related to prolonged inflammation and metabolic disturbances (Table 2).

Laboratory signs of inflammatory activity were observed in both groups. In the clinical remission group, thrombocytosis was present in 17 patients (56.6%), leukocytosis and elevated erythrocyte sedimentation rate were observed in 15 patients (50.0%), and increased C-reactive protein levels were detected in 28 patients (93.3%). In the non-remission group, elevated platelet counts were observed in 21 patients (70.0%), leukocytosis in 19 patients (63.3%), elevated erythrocyte sedimentation rate in 13 patients (43.3%), and increased C-reactive protein levels in 27 patients (90.0%).

Endoscopic examination demonstrated minimal changes, including decreased vascular pattern and contact friability, in 4 patients (13.3%) in the clinical remission group and in 3 patients (10.0%) in the non-remission group. Moderate endoscopic activity, including marked erythema, loss of vascular pattern, erosions, and friability, was observed in 23 patients (76.7%) in the clinical remission group and in 15 patients (50.0%) in the non-remission group. Severe endoscopic activity, characterized by spontaneous bleeding and ulceration, was observed in 3 patients (10.0%) and 12 patients (40.0%), respectively.

**Figure 2:**
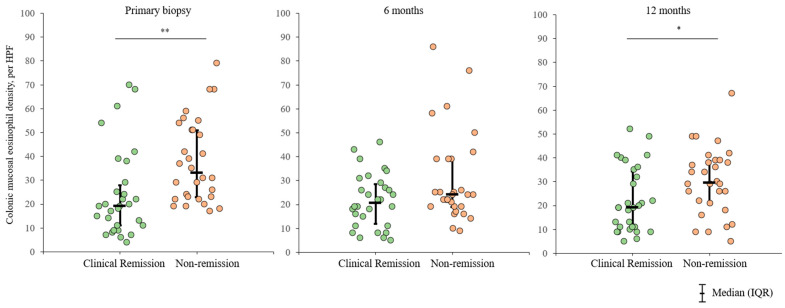
Eosinophil density in the colonic mucosa per microscopic field. Data are presented as median (IQR). Comparisons between groups were performed using the Mann–Whitney U test. * *p* < 0.05; ** *p* < 0.01.

The median eosinophil density in colonic biopsy specimens was 19 (11–27) eosinophils/HPF in the clinical remission group and 33 (23–51) eosinophils/HPF in the non-remission group. The peak eosinophil count was 31 (26–46) and 40 (29–59) cells, respectively (Table 2, Figure 2).

**Figure 3:**
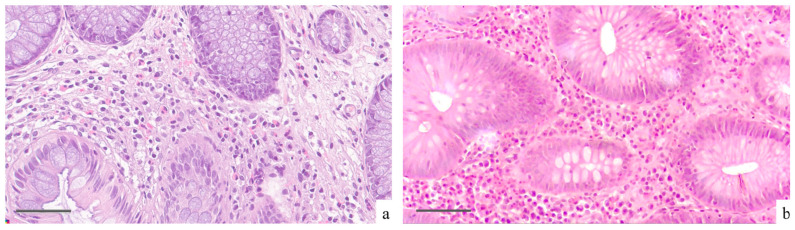
Representative histological sections of primary colonic biopsy specimens from the clinical remission group (**a**) and the non-remission group (**b**). Hematoxylin and eosin staining, ×200, scale bar 60 μm. (**a**) Lymphoplasmacytic inflammation with scattered eosinophils in the lamina propria of the mucosa. (**b**) Marked eosinophilic inflammation in the lamina propria of the mucosa.

Histologically, eosinophils in the clinical remission group were predominantly localized in the lamina propria, forming a moderately pronounced interstitial infiltrate or occurring as scattered individual eosinophils (Figure 3a). In contrast, biopsy specimens from the non-remission group showed marked eosinophilic infiltration of the lamina propria with dense cellular aggregates, predominantly in a pericryptal distribution. In some cases, signs of eosinophil degranulation were also observed (Figure 3b).

### 3.2. Clinical, Laboratory, Endoscopic, and Histological Characteristics of the Study Groups at the 6-Month Follow-Up

At the 6-month follow-up, extraintestinal manifestations were observed in 2 patients (6.7%) in the clinical remission group and in 9 patients (30.0%) in the non-remission group.

In the clinical remission group, most hematological and biochemical parameters were within the reference range. In the non-remission group, elevated platelet counts were observed in 15 patients (50.0%), leukocytosis in 13 patients (43.3%), elevated erythrocyte sedimentation rate in 18 patients (60.0%), and increased C-reactive protein levels in 25 patients (83.3%).

Endoscopic examination demonstrated minimal mucosal changes in all 30 patients (100.0%) in the clinical remission group. In the non-remission group, minimal mucosal changes were observed in 17 patients (56.0%), whereas 13 patients (43.3%) showed moderate endoscopic activity.

The median eosinophil density in colonic biopsy specimens was 21 (12–29) eosinophils/HPF in the clinical remission group and 24 (19–39) eosinophils/ HPF in the non-remission group. The peak eosinophil count was 29 (18–37) cells in both groups (Table 2, Figure 2).

### 3.3. Clinical, Laboratory, Endoscopic, and Histological Characteristics of the Study Groups at the 12-Month Follow-Up

At the 12-month follow-up, extraintestinal manifestations were observed in 2 patients (6.7%) in the clinical remission group and in 9 patients (30.0%) in the non-remission group.

In the clinical remission group, most hematological and biochemical parameters remained within the reference range. In the non-remission group, elevated platelet counts were observed in 14 patients (46.7%), leukocytosis in 11 patients (36.7%), elevated erythrocyte sedimentation rate in 13 patients (43.3%), and increased C-reactive protein levels in 25 patients (83.3%).

Endoscopic examination demonstrated minimal mucosal changes in all 30 patients (100.0%) in the clinical remission group, including reduced vascular pattern and contact friability. In the non-remission group, minimal mucosal changes were observed in 2 patients (6.7%), moderate endoscopic activity in 23 patients (76.7%), and severe endoscopic activity in 5 patients (16.7%).

The median eosinophil density in colonic biopsy specimens was 19 (11–34) eosinophils/ HPF in the clinical remission group and 30 (21–39) eosinophils/ HPF in the non-remission group. The peak eosinophil count was 25 (19–47) and 42 (29–51) cells, respectively (Table 2, Figure 2).

Interobserver agreement for eosinophil density assessment was good (ICC = 0.797; 95% CI 0.730–0.847; *p* < 0.001), and agreement for peak eosinophil count was also good (ICC = 0.652; 95% CI 0.559–0.730; *p* < 0.001). Agreement for the categorical assessment of eosinophilic infiltration was fair (k = 0.491, *p* < 0.001).

### 3.4. Clinical, Laboratory, and Histological Factors Associated with Failure to Achieve Clinical Remission in Ulcerative Colitis

At baseline, extraintestinal manifestations and increased eosinophil density in the colonic mucosa were associated with failure to achieve clinical remission during the 12-month follow-up.

In the univariate analysis, extraintestinal manifestations were associated with higher odds of failure to achieve clinical remission (OR 6.00; 95% CI 1.48–24.30; *p* = 0.012). Eosinophil density and categorical eosinophil assessment were also significantly associated with this outcome (OR 1.045; 95% CI 1.012–1.079; *p* = 0.008 and OR 5.675; 95% CI 1.841–17.494; *p* = 0.003, respectively). The Mayo Endoscopic Subscore and leukocyte count were significant in the univariate model; however, these associations did not remain significant after adjustment.

In the multivariate analysis based on primary biopsy data, extraintestinal manifestations (OR 6.22; 95% CI 1.28–30.16; *p* = 0.023) and eosinophil density (OR 1.04; 95% CI 1.01–1.08; *p* = 0.027) remained statistically significantly associated with failure to achieve clinical remission (Table 3).

When eosinophilic infiltration was analyzed categorically, elevated eosinophil counts were statistically significantly associated with an adverse outcome (OR 6.48; 95% CI 1.76–23.88; *p* = 0.005). Bootstrap analysis confirmed the stability of the effect estimate for eosinophils (*p* = 0.002; 95% CI: 0.602–3.819). Statistical significance was also retained for extraintestinal manifestations (*p* = 0.029); however, the wider confidence interval indicates greater uncertainty of the estimate and lower stability of the effect.

Patients were stratified into four groups according to eosinophilia level (low/high) and histological activity (Nancy Index < 3 and ≥3). Statistically significant differences in the frequency of clinical outcomes were observed among the groups (χ^2^ = 11.41; *p* = 0.010). The highest rate of failure to achieve remission was observed in patients with increased eosinophilia (71.4% in the high eos/low Nancy group and 75.0% in the high eos/high Nancy group), whereas the lowest rate was found in the group with low eosinophilia and low histological activity (23.8%) (Table 4).

In logistic regression analysis, inflammatory phenotypes defined by eosinophilia level and histological activity were statistically significantly associated with outcome (*p* = 0.016). Compared with patients with low eosinophilia and low histological activity, high eosinophilia was associated with higher odds of failure to achieve clinical remission both in the presence of low histological activity (OR = 8.0; 95% CI 1.73–37.09; *p* = 0.008) and elevated histological activity (OR = 9.6; 95% CI 1.85–49.88; *p* = 0.007). Increased histological activity without eosinophilia did not show a statistically significant association with outcome (OR = 2.74; *p* = 0.182).

In the multivariable logistic regression model including eosinophil density, histological activity (Nancy Index), and their interaction term (eos × Nancy), a statistically significant association with adverse outcome was identified only for eosinophilia (OR = 8.0; 95% CI 1.73–37.09; *p* = 0.008). Histological activity (*p* = 0.182) and the interaction term (*p* = 0.479) did not reach statistical significance.

## 4. Discussion

The present study evaluated the potential association of clinical and histological parameters in patients with newly diagnosed ulcerative colitis, with particular emphasis on the role of tissue eosinophilia in the colonic mucosa.

The results of the present study suggest that increased eosinophil density in colonic mucosal biopsies obtained at the time of initial diagnosis is associated with failure to achieve clinical remission during the first year of follow-up. Patients who failed to achieve clinical remission demonstrated significantly higher baseline eosinophil density compared with patients who achieved remission (33 [23,24,25,26,27,28,29,30,31,32,33,34,35,36,37,38,39,40,41,42,43,44,45,46,47,48,49,50,51] vs. 19 [11,12,13,14,15,16,17,18,19,20,21,22,23,24,25,26,27] cells/HPF; *p* = 0.001). Furthermore, elevated tissue eosinophilia was observed in 63.3% of patients in the non-remission group compared with 23.3% in the remission group (*p* = 0.005). Logistic regression analysis showed that increased eosinophil density was associated with failure to achieve clinical remission (OR 6.48; 95% CI 1.76–23.88).

These findings are consistent with accumulating evidence indicating that eosinophils participate in the pathogenesis of inflammatory bowel diseases. Although eosinophils were historically considered mainly as effector cells involved in allergic reactions and parasitic infections, recent studies demonstrate their important role in intestinal immune regulation, epithelial barrier maintenance, and tissue remodeling [14,15,16]. In the intestinal mucosa, eosinophils interact with epithelial cells, macrophages, dendritic cells, and T lymphocytes, contributing to the production of cytokines, chemokines, and lipid mediators that may sustain chronic inflammation [13,16,17,18,19,20,21].

Previous studies investigating the role of tissue eosinophilia in ulcerative colitis have produced heterogeneous results. Some investigators have reported a positive association between mucosal eosinophil density and inflammatory activity or disease severity [22,23,24,25,26,27,28,29,30], while others did not observe a clear relationship between eosinophil infiltration and clinical outcomes [31,32,33,34,35]. In addition, several studies have suggested that eosinophilic infiltration may be associated with an increased risk of relapse in ulcerative colitis [36,37,38,39]. It is important to note that the accumulated evidence suggests distinct roles for eosinophils and neutrophils in ulcerative colitis. While neutrophilic infiltration is closely associated with acute inflammatory activity, epithelial damage, and clinical disease severity, eosinophilia appears to have a more complex and ambiguous relationship with the disease course. Several studies have reported higher eosinophil counts in the context of severe inflammation [55,56], poor response to therapy [20,57] or a higher frequency of relapse [30,58]. On the other hand, some studies have shown that lower eosinophil counts in biopsy specimens were associated with a more severe disease course [28,59] whereas higher eosinophil counts were associated with a better response to therapy [57] or did not correlate with baseline inflammatory severity, treatment response, or relapse [37], and persisted even during the inactive phase of the disease [60]. Taken together, the available evidence suggests that eosinophilia may reflect not so much the intensity of inflammation as the characteristics of an alternative immune response associated with immune regulation, tissue remodeling, and restoration of the epithelial barrier. In this context, increased eosinophilic infiltration may represent a distinct immunoinflammatory phenotype of ulcerative colitis, associated with specific biological mechanisms and potentially different responses to therapy.

Within this conceptual framework, the results of our comparative study showed that a higher level of intestinal eosinophilia in biopsy specimens, including those obtained before the initiation of standard therapy, was associated with failure to achieve clinical remission in the short term. The study design, focused on a clinically more homogeneous subgroup of patients at an early stage of the disease, made it possible to minimize the influence of marked inflammation, severe disease course, and ongoing therapy, all of which may substantially modify the histological picture in ulcerative colitis and thereby mask potential morphological associations. The exclusion of patients receiving biologic therapy was intended to reduce its effect on the cellular composition of the inflammatory infiltrate. The obtained findings suggest that tissue eosinophilia may be associated with specific features of the inflammatory pattern and clinical course of ulcerative colitis.

An important aspect of the present study is that eosinophilic infiltration was evaluated at the time of primary diagnosis, before the long-term effects of therapy on mucosal inflammation could influence the cellular composition of the inflammatory infiltrate. Our findings suggest that increased tissue eosinophilia at an early stage of the disease may be associated with specific features of the inflammatory process in the colonic mucosa.

Interestingly, the number of eosinophils in peripheral blood did not differ significantly between the study groups during the observation period. This observation suggests that the eosinophilic response in ulcerative colitis is largely localized within the intestinal mucosa rather than reflecting systemic eosinophilia, which is consistent with previous observations demonstrating compartmentalization of immune responses in inflammatory bowel diseases [13,45,52].

Another noteworthy observation concerns the temporal dynamics of tissue eosinophilia during follow-up. At baseline, patients who subsequently failed to achieve clinical remission demonstrated significantly higher eosinophil density. At the 6-month follow-up, however, the differences between the groups were no longer statistically significant. By the end of the 12-month observation period, differences in eosinophil density again became apparent. This pattern may reflect partial modulation of the inflammatory infiltrate under the influence of therapy, followed by re-emergence of the underlying inflammatory pattern in patients with persistent disease activity.

In addition to tissue eosinophilia, the present study identified extraintestinal manifestations as another factor associated with failure to achieve clinical remission. Extraintestinal manifestations were significantly more frequent among patients who did not achieve remission (40% vs. 10%; *p* = 0.018). These findings are consistent with previous reports demonstrating that extraintestinal manifestations may occur independently of intestinal inflammatory activity and may reflect a broader systemic inflammatory histopathological pattern in patients with ulcerative colitis [3,61]. However, the wide confidence intervals limit the precision of the estimate and require cautious interpretation.

Taken together, the results of the present study suggest that increased eosinophil density in the colonic mucosa at the time of diagnosis, particularly when combined with the presence of extraintestinal manifestations, may characterize a subgroup of patients with persistent inflammatory activity. Eosinophilia is associated with an adverse outcome regardless of histological activity, which may reflect distinct inflammatory phenotypes in ulcerative colitis.

From a methodological perspective, the study also highlights the importance of quantitative morphometric assessment of eosinophilic infiltration. In the present analysis, the mean eosinophil density demonstrated stronger associations with clinical outcomes than the maximal eosinophil count. This observation suggests that diffuse mucosal eosinophilia may be more informative than isolated focal accumulation of eosinophils. Further studies are required to determine optimal morphometric thresholds for assessing tissue eosinophilia in ulcerative colitis [41,42,43].

In addition, quantitative assessment of eosinophilic infiltration demonstrated higher interobserver reproducibility than categorical classification. These findings highlight the problem of variability in the criteria used to assess tissue eosinophilia in ulcerative colitis and are consistent with the published literature [54,62,63,64,65]. At the same time, the observed agreement estimates may have been partly influenced by the structure of the study sample and require further investigation.

This study has several strengths. First, eosinophil density was assessed in biopsy specimens obtained at the time of initial diagnosis before initiation of therapy, allowing evaluation of the primary inflammatory phenotype. Second, patients were followed longitudinally with repeated endoscopic and histological assessments. Third, morphometric analysis was performed using standardized quantitative methods.

Several limitations should also be acknowledged. The study was conducted at a single center and included a relatively limited number of patients. Despite confirmation of the stability of the observed associations by bootstrap validation, the variability of the estimates and the wide confidence intervals reflect the limited sample size and require cautious interpretation of the effect magnitudes. The study findings reflect histological features of the disease at an early stage and do not allow assessment of its dynamics or long-term course. Segment-specific assessment of eosinophil distribution was not performed. In addition, the retrospective design and the use of a composite endpoint may introduce selection bias and limit the generalizability of the results. An important source of potential systematic bias is the formation of the non-remission group, which included only patients with a partial clinical response, defined as a decrease in the total Mayo score of ≥2 during the first year of follow-up. Patients with a completely refractory disease course or persistently high disease activity without clinical improvement were excluded. Therefore, the non-remission group does not reflect the full spectrum of ulcerative colitis severity. This may have reduced sample heterogeneity and influenced the observed associations. Accordingly, the findings cannot be directly extrapolated to patients with completely refractory or more severe disease and should be interpreted with caution.

## 5. Conclusions

In conclusion, this study suggests that increased eosinophil density in the colonic mucosa at the time of primary diagnosis and the presence of extraintestinal manifestations are associated with failure to achieve clinical remission during the first year of follow-up in patients with ulcerative colitis. The obtained findings suggest a possible association between tissue eosinophilia and clinical outcomes in ulcerative colitis; however, they should be regarded as hypothesis-generating, and prospective multicenter studies involving larger patient cohorts are needed to confirm these observations and clarify their clinical significance.

## Figures and Tables

**Table 1:** Demographic and Clinical Characteristics of Patients.

Characteristics	Total (*n* = 60)	Clinical Remission (*n* = 30)	Non-Remission (*n* = 30)	*p*-Value
Age at diagnosis, years, Median (Q1–Q3)	41.0 (32.0–50.3)	39.5 (31.3–50.8)	41.5 (34.0–50.0)	0.700
Sex, *n* (%)				
Male	34 (56.7)	20 (66.7)	14 (46.7)	0.193
Female	26 (43.3)	10 (33.3)	16 (53.3)	
Race/Ethnicity, *n* (%)				
Asian	35 (58.3)	17 (56.7)	18 (60.0)	0.954
Caucasian	10 (16.7)	5 (16.7)	5 (16.7)	
Other	15 (25.0)	8 (26.7)	7 (23.3)	
BMI, kg/m^2^	23.8 (21.2–25.5)	24.0 (21.6–26.9)	22.9 (20.7–24.8)	0.114
Smoking Status, *n* (%)				
Current smoker	7 (11.7)	3 (10.0)	4 (13.3)	0.310
Former smoker	9 (15.0)	7 (23.3)	2 (6.7)	
Never smoked	29 (48.3)	10 (33.3)	10 (33.3)	
Data not available	33 (55.0)	10 (33.3)	14 (46.7)	
History of Appendectomy, *n* (%)	6 (10.0)	4 (13.3)	2 (6.7)	0.667
Immune-mediated diseases, *n* (%)	8 (13.3)	3 (10.0)	5 (16.7)	0.705
Charlson Comorbidity Index, Median (Q1–Q3)	0.0 (0.0–1.0)	0.0 (0.0–1.0)	0.0 (0.0–1.0)	0.880
Ulcerative Colitis Diagnosis, *n* (%)	60 (100.0)	30 (100.0)	30 (100.0)	-
Time from Symptom Onset to Diagnosis, years, Median (Q1–Q3)	6.0 (1.9–10.0)	5.3 (1.3–9.8)	6.5 (2.6–10.5)	0.382
Disease Extent (Montreal Classification), *n* (%)				
E1 (Proctitis)	9 (15.0)	3 (10.0)	6 (20.0)	0.545
E2 (Left-sided colitis)	29 (48.3)	15 (50.0)	14 (46.7)	
E3 (Extensive colitis/Pancolitis)	22 (36.7)	12 (40.0)	10 (33.3)	
Treatment adherence	60 (0)	30 (100)	30 (100)	-
CMV (PCR, rectum), *n* (%)	0 (0)	0 (0)	0 (0)	-
Colorectal cancer, *n* (%)	0 (0)	0 (0)	0 (0)	-
History of colectomy, *n* (%)	0 (0)	0 (0)	0 (0)	-
Positive Stool for Helminths/Protozoa, *n* (%)	0 (0)	0 (0)	0 (0)	-

**Table 2:** Clinical, Laboratory, and Morphological Characteristics of Patients with Ulcerative Colitis in the Study Groups at Extended Follow-Up.

Characteristics	0 Months (Baseline)			6 Months			12 Months		
	Clinical Remission *n* = 30	Non-Remission *n* = 30	*p*-Value	Clinical Remission *n* = 30	Non-Remission *n* = 30	*p*-Value	Clinical Remission *n* = 30	Non-Remission *n* = 30	*p*-Value
Extraintestinal manifestations, *n* (%)	3 (10.0)	12 (40.0)	0.018	2 (6.7)	9 (30.0)	0.046	2 (6.7)	9 (30.0)	0.046
Laboratory findings									
Platelets, /L	410 (390–472)	460 (397–520)	0.092	287 (252–320)	404 (356–438)	0.000	275 (121–131)	385 (319–412)	0.000
Hemoglobin, g/L	127 (112–134)	119 (107–132)	0.264	129 (124–135)	120 (112–129)	0.006	125 (120–131)	118 (111–121)	0.002
Leukocytes, ×109/L	9.0 (7.3–10.1)	9.7 (8.8–11.2)	0.014	7.8 (7.1–8.5)	8.9 (8.1–9.7)	0.000	7.1 (6,9–7,4)	8.7 (8.4–9.1)	0.000
Eosinophils, ×109/L	0.4 (0.2–0.5)	0.4 (0.3–0.5)	0.114	0.4 (0.3–0.5)	0.4 (0.3–0.5)	0.620	0.4 (0.3–0.5)	0.4 (0.3–0.5)	0.337
ESR, mm/h	20.5 (13–28.3)	22.5 (14.3–31.8)	0.481	16.5 (11.8–20.5)	17 (14–22)	0.371	18 (16–19)	21 (18–23)	0.036
C-reactive protein, mg/L	14.7 (11.5–18.1)	15.4 (9.9–24.9)	0.589	4.2 (3.7–5.0)	10.8 (6.1–19.6)	0.000	5.0 (3.3–5.0)	10.8 (6.1–19.6)	0.000
Fecal calprotectin, IU/mL	654.0 (490.5–994.0)	1537.1 (974.8–1862.4)	0.109	133.0 (84.0–166.8)	494.0 (244–586.5)	0.000	122.5 (89–155.3)	465.0 (341.5–883.3)	0.001
Instrumental examinations									
Mayo Endoscopic Subscore	2 (2–2)	2 (2–3)	0.024	1 (1–1)	2 (1–2)	0.000	1 (1–1)	2 (2–2)	0.000
Total Mayo score	8 (8–9)	9 (8–11)	0.005	1 (1–2)	5 (3.25–6)	0.000	1 (1–2)	6 (5–7)	0.000
Morphological findings									
Nancy Index, *n* (%)									
0	0 (0)	0 (0)	0.183	8 (26.7)	0 (0)	0.000	3 (10.0)	0 (0)	0.001
1	3 (10.0)	1 (3.3)		20 (66.7)	3 (10.0)		16 (53.3)	4 (13.3)	
2	17 (56.7)	14 (46.7)		2 (6.7)	20 (66.7)		11 (36.7)	21 (70.0)	
3	9 (30.0)	9 (30.0)		0 (0)	7 (23.3)		0 (0)	5 (16.7)	
4	1 (3.3)	6 (20.0)		0 (0)	0 (0)		0 (0)	0 (0)	
Biopsy with the most pronounced inflammation, *n* (%)									
Right colon	12 (40.0)	14 (46.7)	0.795	11 (36.7)	14 (46.7)	0.601	10 (33.3)	12 (40.0)	0.789
Left colon	18 (60.0)	16 (53.3)		19 (63.3)	16 (53.3)		20 (66.7)	18 (60.0)	
Eosinophils									
Eosinophil density in the colonic mucosa, per high-power field	19 (11–27)	33 (23–51)	0.001	21 (12–29)	24 (19–39)	0.112	19 (11–34)	30 (21–39)	0.038
Peak eosinophil count	31 (26–46)	40 (29–59)	0.059	29 (18–37)	30 (25–39)	0.245	25 (19–47)	42 (29–51)	0.888
Eosinophils, *n* (%)									
Not elevated	23 (76.7)	11 (36.7)	0.005	27 (90.0)	24 (80.0)	0.470	25 (83.3)	13 (43.3)	0.004
Elevated	7 (23.3)	19 (63.3)		3 (10.0)	6 (20.0)		5 (16.7)	17 (56.7)	
Current therapy, *n* (%)	-	-	-	30 (100)	30 (100)	1.00	30 (100)	30 (100)	1.00
5-ASA	-	-	-	0 (0)	9 (30.0)	0.004	0 (0)	15 (50.0)	0.000
Corticosteroids	-	-	-	0 (0)	13 (43.3)	0.096	0 (0)	11 (36.7)	0.000
Immunomodulators	-	-	-	0 (0)	0 (0)	-	0 (0)	0 (0)	-

**Table 3:** Univariate and Multivariate Logistic Regression Analysis Based on Primary Biopsy Findings.

Variable	Category	Univariate Analysis		Multivariate Analysis	
		OR (95% CI)	*p*-Value	OR (95% CI)	*p*-Value
Quantitative variables					
Extraintestinal manifestations	yes	6.000 (1.482–24.299)	0.012	6.223 (1.284–30.159)	0.023
	no	-	-		
Platelet count, /L	-	0.994 (0.986–1.001)	0.106	-	-
Leukocyte count, ×10^9^/L	-	0.647 (0.464–0.904)	0.011	0.760 (0.511–1.130)	0.175
Mayo Endoscopic Subscore	-	0.335 (0.135–0.934)	0.036	0.415 (0.123–1.397)	0.156
Mean eosinophil density in the colonic mucosa, cells/HPF	-	1.045 (1.012–1.079).	0.008	1.040 (1.004–1.078)	0.027
Qualitative variables					
Extraintestinal manifestations	yes	6.000 (1.482–24.299)	0.012	5.779 (1.169–28.561)	0.031
	no	-	-		
Platelet count, /L	-	0.994 (0.986–1.001)	0.106	-	-
Leukocyte count, ×10^9^/L	-	0.647 (0.464–0.904)	0.011	0.725 (0.480–1.093)	0.125
Mayo Endoscopic Subscore	-	0.335 (0.135–0.934)	0.036	0.336 (0.089–1.267)	0.107
Eosinophils	Not elevated	5.675 (1.841–17.494)	0.003	6.480 (1.759–23.878)	0.005
	Elevated				

**Table 4:** Clinical outcomes according to inflammatory phenotypes.

Groups *	Clinical Remission (*n* = 30)	Non-Remission (*n* = 30)	*p*-Value
Low eos/Low Nancy	16 (76.2%)	5 (23.8%)	χ^2^ = 11.41 0.010
High eos/Low Nancy	4 (28.6%)	10 (71.4%)	
Low eos/High Nancy	7 (53.8%)	6 (46.2%)	
High eos/High Nancy	3 (25.0%)	9 (75.0%)	

* Eosinophil density was categorized as low or high using previously proposed cutoff values: ≥50 eosinophils per high-power field (HPF) in the right colon and ≥25 eosinophils per HPF in the left colon were defined as high eosinophilia, whereas values below these thresholds were considered low eosinophilia. Histological activity was defined as low (Nancy Index < 3) or high (Nancy Index ≥ 3). Pearson’s χ^2^ test.

## Data Availability

Data are available upon reasonable request to the authors.
